# Pharmacokinetic Evaluation of GB-5001, a Long-Acting Injectable Formulation of Donepezil, in Healthy Korean Participants: Population Pharmacokinetics with Phase 1 Study

**DOI:** 10.3390/pharmaceutics17121517

**Published:** 2025-11-25

**Authors:** Ye Chan Park, Eunyoung Seol, Jongmi Lee, Jang Hee Hong, Jin-Gyu Jung, Jung Sunwoo

**Affiliations:** 1 Clinical Trials Center, Chungnam National University Hospital, Daejeon 35015, Republic of Korea; dpcks33@naver.com (Y.C.P.); boniii@cnu.ac.kr (J.H.H.); 2 Department of Medical Science, College of Medicine, Chungnam National University, Daejeon 35015, Republic of Korea; 3 R&D Center, G2GBIO, Inc., CheongJu 28160, Republic of Korea; eunyoung.seol@g2gbio.com (E.S.); jongmi.lee@g2gbio.com (J.L.); 4 Department of Pharmacology, College of Medicine, Chungnam National University, Daejeon 35015, Republic of Korea; 5 Department of Family Medicine, College of Medicine and Hospital, Chungnam National University, Daejeon 35015, Republic of Korea; jjg7272@gmail.com

**Keywords:** pharmacokinetics, pharmacodynamics, safety, tolerability, donepezil, long-acting formulation, GB-5001, long-acting injectable, Alzheimer’s disease, phase 1

## Abstract

**Background/Objectives**: Oral donepezil, an acetylcholinesterase (AChE) inhibitor for Alzheimer’s disease, faces adherence challenges. Long-acting injectable (LAI) formulations like GB-5001 aim to enhance adherence by reducing dosing frequency. This Phase 1, open-label, active-controlled, dose-escalation study evaluated the safety, tolerability, pharmacokinetics (PK), and pharmacodynamics (PD) of GB-5001 in healthy male adults. **Methods**: Participants were assigned to cohorts receiving GB-5001A or GB-5001D (LAI formulations) via intramuscular (IM) or subcutaneous (SC) injection, or oral Aricept^®^. Safety, PK, and PD (AChE inhibition) were assessed. The influence of CYP2D6 phenotype was explored, and modeling/simulation was performed. **Results**: Fifty healthy male participants completed the study. After IM administration, GB-5001A (70 mg, 140 mg, 280 mg) showed dose-dependent increases in exposure (AUC_inf_ and C_max_), resulting in significantly extended exposure compared to oral Aricept^®^ 10 mg. No serious adverse events were reported; the most common AEs were mild injection site reactions, which occurred in all treatment groups except the GB-5001A IM 70 mg group and the Aricept group. GB-5001A also demonstrated sustained AChE inhibition. **Conclusions**: GB-5001A, an LAI donepezil, showed favorable safety, dose-proportional PK, and sustained plasma exposure. It achieved a 3–4-fold longer half-life than oral donepezil. These findings, supported by modeling, highlight GB-5001A’s potential as a once-monthly IM alternative for Alzheimer’s disease treatment.

## 1. Introduction

Dementia is characterized by a gradual deterioration of cognitive functions, including memory, reasoning, and judgment, to a degree that significantly impairs independent daily living. Affected individuals may also exhibit emotional dysregulation and alterations in personality. The condition progresses from mild cognitive impairment, which may subtly affect daily routines, to severe stages requiring complete assistance with fundamental activities such as feeding and personal care [1]. Alzheimer’s disease, the most common form of dementia, is pathologically characterized by the accumulation of β-amyloid plaques and the hyperphosphorylation of tau protein, leading to the formation of neurofibrillary tangles; these proteinopathies disrupt neuronal function and connectivity, ultimately resulting in synaptic loss and neurodegeneration [2].

In 2021, approximately 57 million people worldwide were living with dementia, with over 60% of cases occurring in low- and middle-income countries. Each year, close to 10 million new cases are identified [3].

As of 2024, caregivers of individuals with Alzheimer’s disease or other forms of dementia are estimated to have provided 19.2 billion hours of unpaid care, which is valued at approximately $413.5 billion. The estimated lifetime cost of care for a person with dementia in 2024 is approximately $405,262, with 70% of this cost borne by family caregivers in the form of unpaid care and out-of-pocket expenses [4]. Additionally, the global direct and indirect costs of dementia are projected to surge from $1.33 trillion in 2020 to $9.12 trillion by 2050 [5].

Donepezil is an acetylcholinesterase inhibitor that was first developed in 1996 under the brand name Aricept^®^ and has been widely used for the treatment of Alzheimer ’s-type dementia. Currently, it is administered once daily at doses of 5 mg or 10 mg for patients with mild to moderate Alzheimer’s disease, and at doses of 10 mg or 23 mg for those with moderate to severe disease [6]. Following the development of the oral formulation, alternative delivery methods were explored to bypass the first-pass effect, enhance convenience, and support self-administration. Although a transdermal formulation was introduced in 2010, it was later withdrawn by the U.S. FDA. More recently, in 2022, a new donepezil transdermal system was developed and received FDA approval [7,8].

Donepezil is predominantly metabolized in the liver, producing multiple metabolites, while both the parent drug and its metabolites are cleared mainly through renal excretion [9]. Its metabolic conversion is mediated primarily by the cytochrome P450 isoenzymes CYP3A4 and CYP2D6 [10]. Among the identified metabolites, 6-O-desmethyl donepezil is regarded as the major active metabolite and has been reported to possess cholinesterase inhibitory activity comparable to that of the parent compound [11].

Cognitive impairment has been reported to affect a patient’s ability to manage medications. It can lead to situations where patients are unable to open medication containers or refuse to take their medications, resulting in decreased adherence. Consequently, the responsibility of medication management can shift from the patient to a caregiver. This shift, along with the cognitive decline caused by dementia, has been shown to impact medication adherence depending on the severity of the condition [12].

The impact of medication adherence and disease control is well recognized. Long-acting injectable (LAI) formulations convert the daily oral administration of medications into a single injection, thereby significantly improving convenience by reducing the frequency of dosing over several days or weeks. LAI formulations have been shown to improve adherence compared to oral medications, as patients receiving LAIs are more likely to maintain consistent medication use and experience lower rates and risk of treatment discontinuation [13].

To mitigate challenges commonly associated with conventional dosing regimens, particularly suboptimal medication adherence and its impact on effective disease management, the development of an LAI formulation of donepezil may represent a promising therapeutic advancement in the treatment of Alzheimer’s disease. GB-5001, a once-monthly intramuscular (IM) formulation of donepezil, was developed to address these limitations. This study aimed to evaluate the safety and tolerability of GB-5001, as well as the pharmacokinetics (PK) of both donepezil and its active metabolite 6-O-desmethyl donepezil, in healthy adults. In addition, exploratory pharmacodynamic (PD) assessments and pharmacokinetic PK simulations following multiple dosing were conducted as foundational steps toward its potential application in patients with mild to severe Alzheimer’s disease.

## 2. Materials and Methods

### 2.1. Ethics

**Figure 1:**
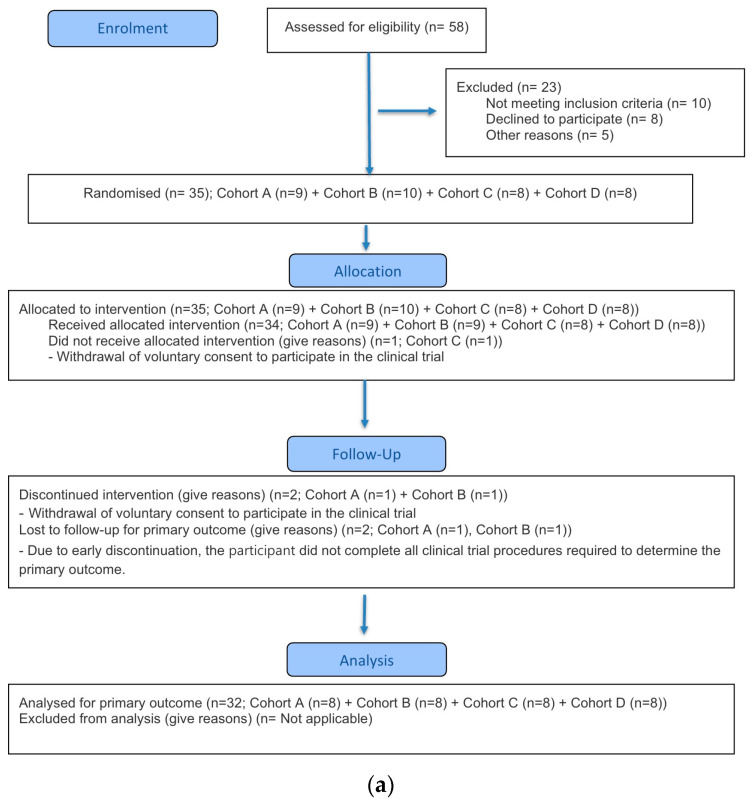

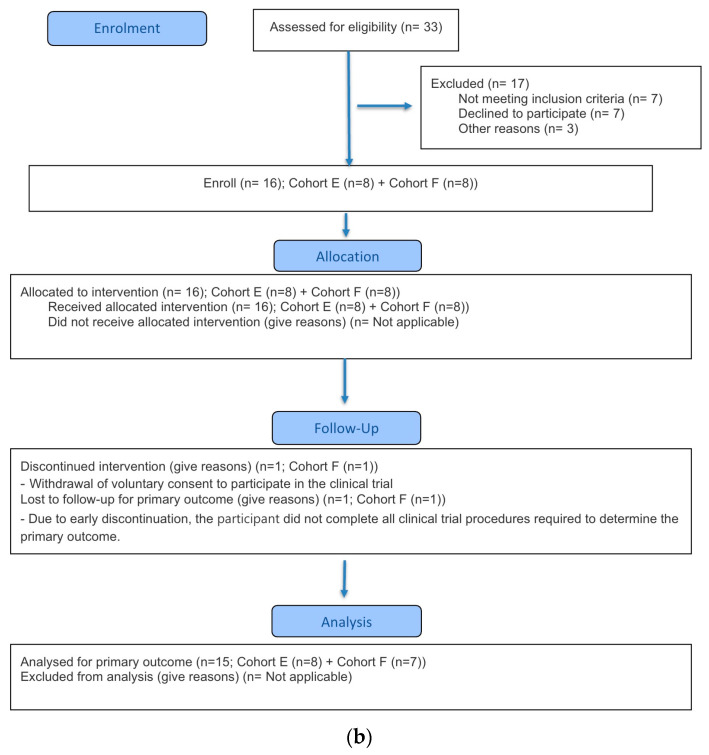
CONSORT flow diagram. (**a**) Part A (**b**) Part B.

This clinical trial was conducted in accordance with the Good Clinical Practice guidelines established by the International Council for Harmonisation (ICH-GCP) and the ethical principles outlined in the Declaration of Helsinki. The study protocol was approved by the Korea Ministry of Food and Drug Safety and the Institutional Review Board (IRB) of Chungnam National University Hospital (approval number: CNUH 2023-08-088; approval date: 12 October 2023). This study was conducted in compliance with the CONSORT guidelines (Figure 1). The trial was registered with ClinicalTrials.gov (NCT06127368, approval date 7 November 2023).

### 2.2. Study Design

This open-label, active-controlled, parallel-group, dose-escalation phase 1 clinical trial was conducted at the Clinical Trial Center of Chungnam National University Hospital from the first participant enrollment on 3 January 2024, to the last participant’s end-of-observation on 16 October 2024. The trial consisted of two parts, Part A and Part B. Part A included four cohorts, including an active control group (Aricept^®^ 10 mg tablet). Cohorts A and B received 70 mg of GB-5001A (donepezil 70 mg) via IM and subcutaneous (SC) injection, respectively. Cohort C received 70 mg of GB-5001D (donepezil 70 mg) via SC injection, and Cohort D received Aricept^®^ 10 mg orally. Part B included Cohorts E and F, which received 140 mg and 280 mg of GB-5001A via IM injection, respectively.

### 2.3. Investigational Products

Both GB-5001A and GB-5001D are LAI formulations of donepezil developed by G2GBIO (Cheongju, Republic of Korea) and were used as investigational products in this clinical trial. GB-5001A was administered via both IM and SC routes at a dose of 70 mg/0.55 mL in Cohorts A and B, and IM route for 140 mg/1.1 mL and 280 mg/2.2 mL in Cohorts E and F, respectively. GB-5001D was administered exclusively via the subcutaneous route at a dose of 70 mg/0.4 mL in Cohort C, as preclinical studies conducted prior to this clinical trial demonstrated that the SC route provided a PK profile more suitable for LAI delivery compared with IM administration. All IM injections were administered into the right ventrogluteal area, while SC injections were given in the right abdominal area. Participants were instructed to avoid applying pressure or massaging the injection site after administration to prevent any interference with drug absorption.

### 2.4. Participants

All participants voluntarily consented to participate in the study through a written informed consent process approved by the IRB. Healthy Asian adult male volunteers aged between 19 and 55 years, with a body weight of at least 55 kg and a body mass index between 18.5 kg/m^2^ and 30.0 kg/m^2^, were enrolled. Eligible participants met all inclusion criteria and had no relevant medical history, symptoms, viral infections, alcohol abuse or dependence, or drug abuse or dependence. In addition, all participants had normal results in clinical laboratory tests, physical examination, and vital signs assessments at screening. For Part B of the study, participants identified as poor metabolizers based on Cytochrome P450 2D6 (CYP2D6) phenotyping were excluded because of the potential risk of substantially increased exposure and prolonged elimination of donepezil following administration of the long-acting injectable formulation.

### 2.5. Study Objectives

The primary objective of this study was to evaluate the safety and tolerability of a single administration via IM or SC routes of long-acting donepezil formulations. The secondary objective included the assessment of the PK parameters of 6-O-desmethyl donepezil, an active metabolite of donepezil, following single IM and SC administration of GB-5001 and oral administration of Aricept^®^. Additionally, for exploratory purposes, the PD of donepezil was assessed after a single IM administration of GB-5001A. Furthermore, pharmacokinetic modeling and simulation were conducted based on the single administration of GB-5001A via IM route and oral administration of Aricept^®^, with the aim of predicting the pharmacokinetics of GB-5001A following repeated administration.

### 2.6. Pharmacokinetic and Pharmacodynamic Parameters

In this study, to evaluate the PK of donepezil, the following parameters were assessed after single-dose administration of GB-5001A, GB-5001D, and Aricept^®^: area under the blood concentration–time curve from time zero to the time of the last quantifiable concentration (AUC_last_), area under the blood concentration–time curve from time zero extrapolated to infinity (AUC_inf_), area under the blood concentration–time curve from time zero to 720 h (AUC_0–720_), maximum concentration of drug (C_max_), apparent clearance (CL/F), volume of distribution (V_d_/F), half-life (t_1/2_), time to maximum concentration (T_max_), and time prior to the first measurable concentration (T_lag_). For the active metabolite, 6-O-desmethyl donepezil, PK parameters including AUC_last_, AUC_inf_, AUC_0–720_, C_max_, CL/F, V_d_/F, t_1/2_, T_max_, and T_lag_ were assessed following the administration of GB-5001A at 280 mg. For PD evaluation, E_max_ was defined as the maximum observed AChE inhibition (%), the area under the effect curve from time zero to the time of the last quantifiable activity (AUE_last_) and E_max_ were assessed after single-dose administration of GB-5001A at 280 mg.

In our study, drug exposure was evaluated using three AUC-based pharmacokinetic parameters. AUC_0–720_ was selected as a parameter reflecting the long-acting characteristics of GB-5001A, allowing assessment of early exposure over the first 30 days after dosing. AUC_last was used to evaluate drug exposure based on the concentrations measured in our study, while AUC_inf_ was used to characterize the total systemic exposure not fully captured by AUC_last_.

In the modeling and simulation analysis, pharmacokinetic parameters of simulated donepezil profiles were evaluated, including the C_max_ at steady-state (C_max,ss_), minimum concentration at steady-state (C_min,ss_), AUC over the dosing interval (AUC_τ_), average concentration at steady-state (C_av,ss_), and fluctuation index (Swing_tau).

### 2.7. Blood Sampling and Preparation

PK and PD analyses were performed using blood samples collected after donepezil administration. All analyses were conducted based on the scheduled blood sampling time points. For PK sampling, Cohorts A, B, E, and F followed the schedule of blood collection at pre-dose (0 h) and at 0.5 h, 1 h, 2 h, 4 h, 6 h, 8 h, 12 h, 24 h (D2), 48 h (D3), 72 h (D4), 96 h (D5), 120 h (D6), 168 h (D8), 240 h (D11), 312 h (D14), 360 h (D16), 432 h (D19), 504 h (D22), 552 h (D24), 600 h (D26), 648 h (D28), 720 h (D31), 768 h (D33), 840 h (D36), 1008 h (D43), 1176 h (D50), 1344 h (D57), 1512 h (D64), 1848 h (D78), 2184 h (D92) and 2352 h (D99) post-dose. Cohort C followed the same sampling schedule as these cohorts but ended at 1512 h (D64). Cohort D underwent a more intensive early sampling schedule up to 240 h (D11), including frequent timepoints within the first 6 h after dosing (Table S1). PD analysis was conducted only in Cohort F using the same blood samples collected for PK analysis.

For PK analysis, blood samples were centrifuged (Combi 514R; Hanil Scientific, Gimpo, Gyeonggi-do, Republic of Korea) within 1 h of collection at 600× g for 10 min at 4 °C to separate plasma. The plasma samples were stored at or below −70 °C in a deep freezer (TDE60086FD-ULTS; Thermo Fisher Scientific, Waltham, MA, USA) until analysis.

For PD analysis, 2 mL of red blood cells (RBCs) from the bottom layer of the centrifuged blood collection tube (used for PK) were transferred into a 15 mL conical tube. Sterile normal saline (0.9%) was added at three times the volume of the RBCs (total 6 mL), and the sample was gently inverted five times. This mixture was centrifuged at 600× *g* for 10 min at 4 °C, and the supernatant was discarded. This washing procedure was repeated twice. After the second centrifugation, the supernatant was removed, and the remaining RBCs were stored at or below −70 °C in a deep freezer until PD analysis.

### 2.8. Pharmacokinetics and Pharmacodynamics Analysis

The plasma concentrations of donepezil and its active metabolite, 6-O-desmethyl donepezil, were measured using a validated liquid chromatography–tandem mass spectrometry (LC-MS/MS) method for PK analysis. Concentration values below the quantifiable limit (BQL) were handled as follows: if BQL values occurred prior to T_max_, they were treated as zero; if they occurred after T_max_, they were considered as ‘blank’ and excluded from the analysis. Pharmacokinetic parameters were calculated using the non-compartmental method of Phoenix WinNonlin^®^ version 8.5 (Certara, L.P., Princeton, NJ, USA). C_max_ and T_max_ were determined from observed values, while the AUC was calculated using the linear trapezoidal method.

For PD analysis, the acetylcholinesterase (AChE) inhibitory activity was measured using an absorptiometric method with the acetylcholinesterase activity assay kit (SIGMA-ALDRICH^®^, St. Louis, MO, USA). Stock solutions included in the kit were used, and 2 mg of reagent was weighed into a tube and reconstituted with assay buffer to prepare a 10 mg/mL working solution. The stability requirement for the reagent was to use the freshly prepared working solution within 30 min, and in this study, all working solutions were used within this 30 min window. In addition, no reconstituted reagent was stored, and all remaining solution was discarded to ensure stability.

For assay controls, water and the calibrator (200 U/L) provided in the kit were used and processed using the same sample preparation procedure. For the RBC samples, 10 µL of sample was added to a tube, followed by 40 µL of assay buffer, and vortexed. Subsequently, 10 µL of the diluted sample was transferred to a new tube, and 70 µL of assay buffer was added and vortexed again. Analyses prioritized measurements within the linear calibration range (10–600 units/L); if a value exceeded 600 units/L, the dilution factor was adjusted accordingly for analysis.

The enzymatic reaction was measured by loading water, calibrators, and processed samples onto a 96-well flat-bottom plate in duplicate (200 µL and 10 µL/well). The percentage change from baseline in AChE inhibitory activity was calculated, and PD parameters were estimated using the non-compartmental method in Phoenix WinNonlin^®^ version 8.5. (Certara, L.P., Princeton, NJ, USA) Emax was determined from observed values, and AUE was calculated using the linear trapezoidal method.

### 2.9. Safety and Tolerability Analysis

In this study, the following safety assessments were collected and comprehensively reviewed for evaluation: adverse event (AE), adverse drug reaction (ADR), serious AE/ADR (SAE/SADR), unexpected ADR (UADR), injection site reactions (ISRs), concomitant medication and prior medication history, vital signs (blood pressure, pulse rate, and tympanic temperature), physical examination findings, clinical laboratory test results, and 12-lead electrocardiogram (12-lead ECG). Tolerability was evaluated using the data collected for safety assessment.

### 2.10. Pharmacokinetic Modeling and Simulation

Pharmacokinetic modeling and simulation in this study were conducted using data from participants who received GB-5001A via IM administration and those who received Aricept^®^ 10 mg via oral administration, incorporating individual-level covariates such as sex, age, body weight, and CYP2D6 metabolizer phenotype. NONMEM^®^ version 7.5 (ICON plc, Dublin, Ireland) was used for the pharmacokinetic modeling and simulation. The model development process included the selection of a basic structural model, incorporation of random effects, and establishment of a full model.

**Figure 2:**
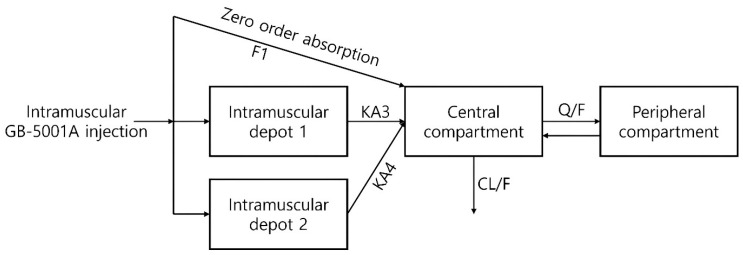
Basic structural model for GB-5001A population pharmacokinetic modeling and simulation. Structural model describing the pharmacokinetic disposition of GB-5001A following intramuscular administration. The model includes two intramuscular depot compartments, from which drug absorption into the central compartment occurs via first-order processes (KA3 and KA4), as well as a zero-order absorption pathway defined by the fraction absorbed (F1). The central compartment is connected to a peripheral compartment through intercompartmental clearance (Q/F), and systemic clearance is represented by CL/F. Arrows represent the modeled structural flow (absorption, distribution, and elimination) and the associated pharmacokinetic parameters.

To identify an appropriate compartmental structure, pharmacokinetic model exploration was performed. For GB-5001A, a two-compartment model with three distinct absorption pathways was assumed. The model comprised a central and a peripheral compartment, with absorption into the central compartment occurring via one zero-order absorption and two first-order absorptions from an intramuscular depot (Figure 2). For orally administered Aricept^®^ 10 mg, a conventional two-compartment model with first-order absorption was used (Figure S1).

In the basic structural model used in this study, unexplained variability not accounted for by inter-individual variability was modeled using a combined error model. A single residual error term (*ε*) was applied to the predicted value (*Y*), with weights assigned based on a proportional component (*θ_1_*) and an additive component (*θ_2_*), thereby implementing a combined error structure (Equation (1)).

(1) $$Y=F+\sqrt{F^{2}\;*\;\theta_{1}^{2}\;*\;\theta_{2}^{2}\;*\;\varepsilon },\;\;\;\;\;\;\;\;\;\;\;\;\;\;\;\;\;\;\;\;\;\;\varepsilon \sim N(0,\;1^{2})$$

The intercompartmental clearance (Q) in GB-5001A model was fixed at 100 L/h in the final model. Initial attempts to estimate Q resulted in model instability. This was attributed to the flip-flop kinetics of the formulation and the limited sampling points available to sufficiently characterize the initial distribution phase. The fixed value of 100 L/h was selected based on preliminary estimation results from the current dataset, while also taking into consideration the estimated value (Q = 185 L/h) reported in a previous study of the earlier formulation [14].

**Figure 3:**
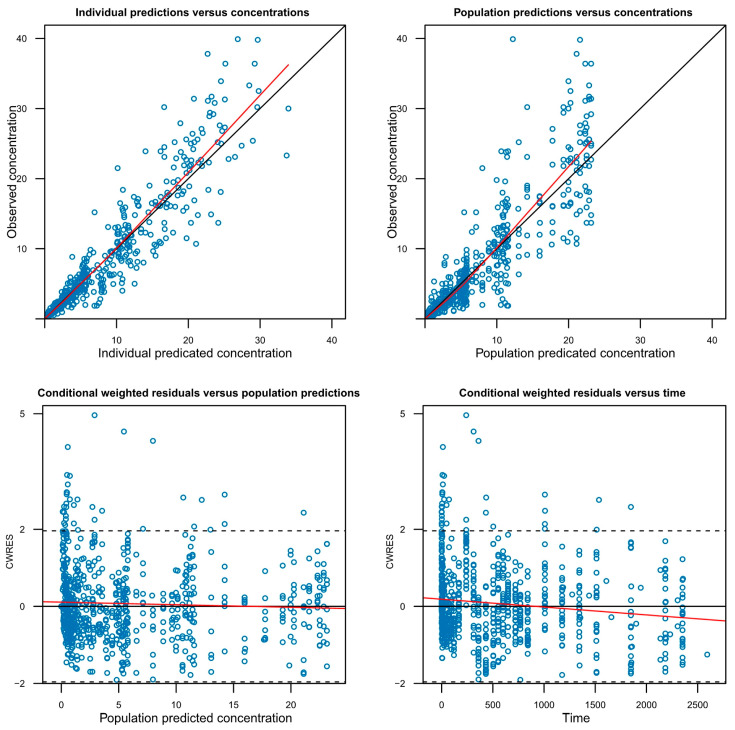
Goodness-of-fit plots for the final GB-5001A pharmacokinetic model. The plots display the agreement between observed and predicted concentrations at the individual (Individual predictions versus concentrations) and population (Population predictions versus concentrations) levels, as well as the conditional weighted residuals (CWRES) versus population predictions (Conditional weighted residuals versus population predictions) and versus time (Conditional weighted residuals versus time). The red lines represent locally weighted scatterplot smoothing, and the solid black line in the top panels indicates the line of identity. The horizontal dashed lines represent 2 standard deviations.

A successful full model was defined as one in which minimization was successfully completed while including as many covariates as possible that were potentially significant during the covariate screening process. However, models with covariance step failure or rounding errors were not excluded from full model establishment, as these issues do not necessarily indicate model misspecification. To evaluate the adequacy of the model, graphical goodness-of-fit plots were utilized (Figure 3, Figure S2) and a visual predictive check (VPC) was performed (Figure S3).

### 2.11. Dose Proportionality Evaluation

Dose proportionality was evaluated in Cohorts A, E, and F, in which participants received GB-5001A at doses of 70 mg, 140 mg, and 280 mg, respectively. All cohorts received the same investigational product via the same IM route of administration. To assess dose proportionality, a linear regression analysis was performed using SAS^®^ (version 9.4, SAS Institute Inc., Cary, NC, USA), with the log-transformed PK parameters (AUC_last_, AUC_inf_, and C_max_) as dependent variables and the log-transformed dose as the independent variable. The slope and its 95% confidence interval (CI) were estimated. In addition, log-transformed dose-normalized AUC_last_, AUC_inf_, and C_max_ values were analyzed using analysis of variance (ANOVA) to test for differences among cohorts at a significance level of 0.05.

### 2.12. Statistical Analysis

Statistical analyses were performed using SAS^®^ (version 9.4, SAS Institute Inc., Cary, NC, USA), with a two-sided significance level set at 5%. All *p*-values were rounded to four decimal places; *p*-values less than 0.001 were presented as “<0.0001.” For continuous variables, descriptive statistics were presented for each cohort. When the assumption of normality was met, ANOVA was used. If the normality assumption was not satisfied, the Kruskal–Wallis test was applied. For categorical variables, descriptive statistics were also presented by cohort, and the Chi-square test was used to evaluate differences among cohorts. If more than 20% of the cells had expected counts less than or equal to 5, Fisher’s exact test was applied instead.

AEs were coded using MedDRA version 27.1 or higher and were summarized by System Organ Class (SOC) and Preferred Term (PT). The number and percentage of participants experiencing each AE, the number of occurrences, and the 95% CIs for the incidence rates were reported. If a single participant experienced multiple AEs within the same category, the participant was counted once for that category. However, participants could be counted in more than one category if they experienced AEs in multiple categories.

## 3. Results

### 3.1. Study Population and Demographics

A total of 91 participants were screened for study participation, of whom 40 participants failed the screening. In Part A, 35 participants were enrolled, and 34 participants were randomized and received the investigational product (IP), excluding one participant in Cohort B who withdrew prior to dosing. After IP administration, one participant from Cohort A and one from Cohort B discontinued the study, resulting in 32 participants (8 in each cohort) completing the study. In Part B, 16 participants were enrolled, and all were randomized and received the IP without any discontinuation prior to dosing. Following IP administration, one participant in Cohort F discontinued, and a total of 15 participants completed the study (Cohort E: 8; Cohort F: 7).

Participant demographics for each part were analyzed based on those who were randomized and received the IP. In Part A, a total of 34 male participants were included in the demographic analysis set. The mean ± standard deviation (±SD) age was 29.53 ± 6.43 years, with 29.22 ± 6.82 years in Cohort A (*n* = 9), 26.78 ± 4.76 years in Cohort B (*n* = 9), 30.25 ± 5.47 years in Cohort C (*n* = 8), and 32.25 ± 8.19 years in Cohort D (*n* = 8). The mean ± SD body weight in Part A was 73.31 ± 8.72 kg, with 74.28 ± 8.90 kg in Cohort A, 70.00 ± 7.54 kg in Cohort B, 74.74 ± 10.75 kg in Cohort C, and 74.51 ± 8.25 kg in Cohort D.

In Part B, all 16 participants included in the demographic analysis set were also male. The mean ± SD age was 31.00 ± 8.10 years, with 30.75 ± 10.21 years in Cohort E (*n* = 8) and 31.25 ± 6.02 years in Cohort F (*n* = 8). The mean ± SD body weight was 71.83 ± 10.43 kg, with 72.69 ± 13.39 kg in Cohort E and 70.98 ± 7.21 kg in Cohort F. Further detailed demographic information is presented in Table 1.

### 3.2. Pharmacokinetics

In this study, PK analysis was conducted on participants who received the IP, had no major protocol deviations, and completed all scheduled PK sampling. For participants who discontinued early, data collected up to the time of discontinuation were reviewed.

### 3.3. Donepezil

**Figure 4:**
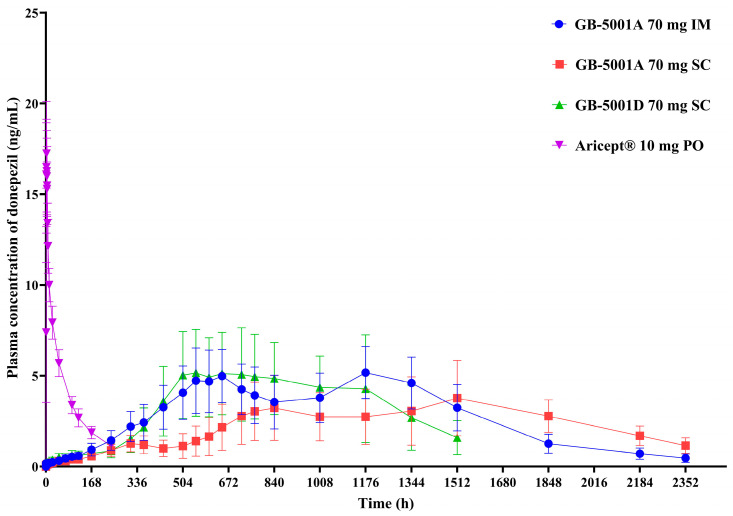
Mean plasma concentration of donepezil in Part A. Mean (±standard deviation) plasma concentration–time profiles of donepezil following administration of GB-5001A 70 mg via IM injection (●), GB-5001A 70 mg SC injection (■), GB-5001D 70 mg SC injection (▲), and oral Aricept^®^ 10 mg (▼) in healthy participants.

In Part A, the GB-5001A 70 mg IM group showed the highest donepezil exposure, with an AUC_last_ (95% CI) of 6991.81 (4694.41–7978.01) h·ng/mL. This group also had the highest C_max_ (95% CI), measured at 5.28 (4.06–6.87) ng/mL. Pharmacokinetic parameters for the other Part A groups are presented in Table 2 (Figure 4).

**Figure 5:**
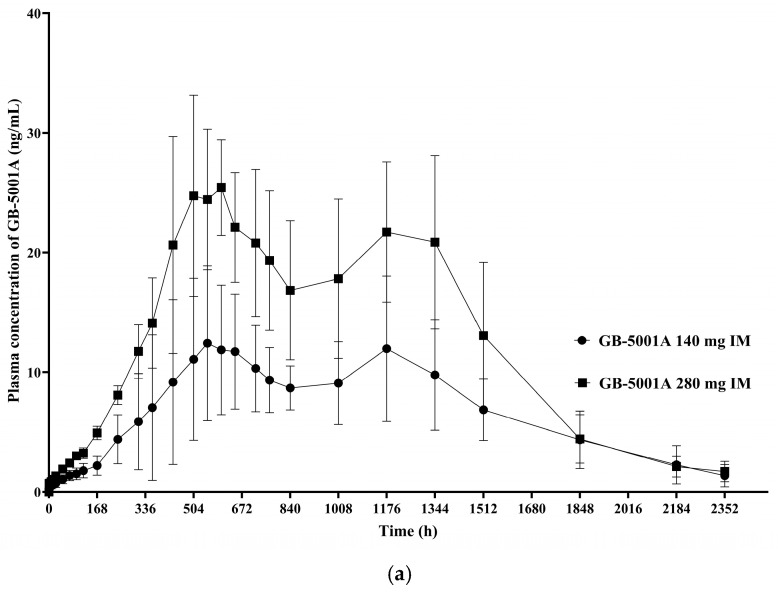

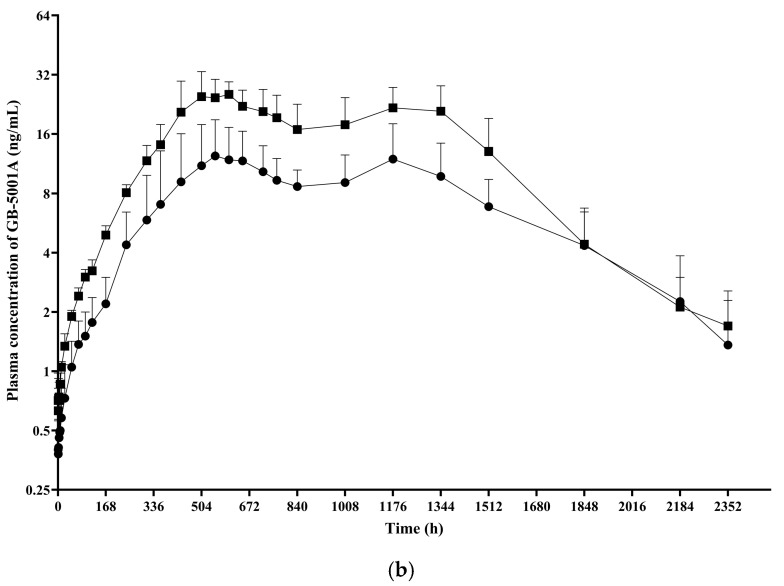
Mean plasma concentration of GB-5001A in Part B. Mean (±standard deviation) plasma concentration–time profiles of GB-5001A following IM administration of 140 mg (●) and 280 mg (■) in healthy participants. (**a**) Linear scale. (**b**) Semi-logarithmic scale.

In Part B, the GB-5001A 280 mg IM group exhibited the greatest donepezil exposure, with an AUC_last_ (95% CI) of 28,981.27 (24,212.79–34,688.86) h·ng/mL and a C_max_ (95% CI) of 29.12 (23.66–35.84) ng/mL. Pharmacokinetic parameters for the other Part B group is presented in Table 2. (Figure 5).

### 3.4. 6-O-Desmethyl Donepezil

**Figure 6:**
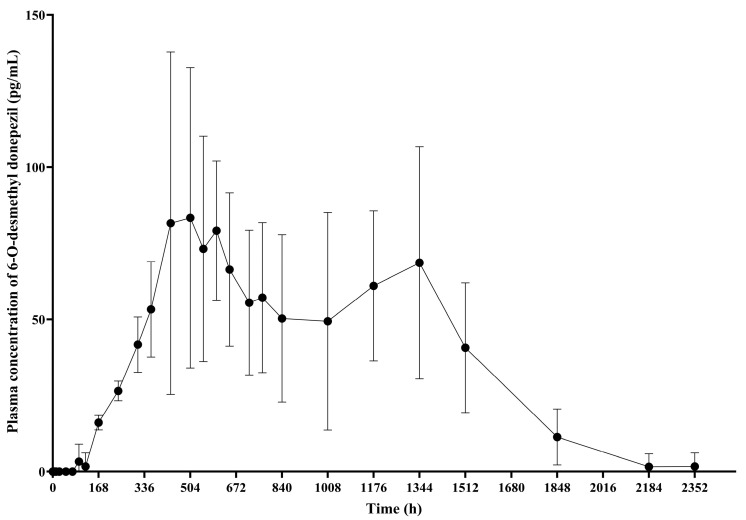
Mean plasma concentration of 6-O-desmethyl donepezil. This graph presents the plasma concentration–time profiles of 6-O-desmethyl donepezil following IM administration of GB-5001A 280 mg, expressed as the mean ± standard deviation.

PK analysis of 6-O-desmethyl donepezil, the active metabolite of donepezil, was conducted exclusively in the GB-5001A 280 mg IM group. The geometric mean (95% CI) of AUC_last_ was 80,078.69 (54,625.31–117,392.42) h·ng/mL, AUC_inf_ was 85,087.25 (58,899.81–122,917.90) h·ng/mL, AUC_0–720_ was 31,156.10 (22,682.70–42,794.84) h·ng/mL, and C_max_ was 100.12 (66.73–150.20) ng/mL, t_1/2_ was 223.16 (178.41–279.14) h, and the median T_max_ was 552.03 h, with a range of 432.03 to 1344.00 h (Table S2, Figure 6).

### 3.5. Pharmacodynamics

PD analysis of donepezil, based on AChE inhibition, was conducted using data from participants in Cohort F. The geometric mean (95% CI) AUE_last_ was 52,373.11 (41,037.97–66,839.15) h, and E_max_ was 37.06 (28.94–47.44%) % (Table 3).

### 3.6. Safety and Tolerability

Participants who received at least one dose of the IP were included in the safety analysis set, comprising 34 participants in Part A and 16 in Part B.

In Part A, the incidence of AEs was 73.53% (25 of 34 participants, 60 events), with cohort-specific incidences of 77.78% (7/9 participants, 18 events) in GB-5001A 70 mg IM group, 88.89% (8/9 participants, 21 events) in GB-5001A 70 mg SC group, 100.00% (8/8 participants, 18 events) in GB-5001D 70 mg SC group, and 25.00% (2/8 participants, 3 events) in Aricept^®^ 10 mg PO group. The incidence of ADRs was 50.00% (17 of 34 participants, 31 events), observed in 88.89% (8/9 participants, 13 events) in GB-5001A 70 mg SC group, 100.00% (8/8 participants, 16 events) in GB-5001D 70 mg SC group, and 12.50% (1/8 participants, 2 events) in Aricept^®^ 10 mg PO group; no ADRs were reported in GB-5001A 70 mg IM group. No serious AEs or serious ADRs occurred, and no participants discontinued due to AEs.

In Part B, the AE incidence was 87.50% (14 of 16 participants, 31 events), with 75.00% (6/8 participants, 12 events) in GB-5001A 140 mg IM group and 100.00% (8/8 participants, 19 events) in GB-5001A 280 mg IM group. The incidence of ADRs was 81.25% (13 of 16 participants, 19 events), reported in 75.00% (6/8 participants, 8 events) in GB-5001A 140 mg IM group and 87.50% (7/8 participants, 11 events) in GB-5001A 280 mg IM group. No serious AEs or ADRs were reported, and no withdrawals occurred due to AEs.

When AEs in Part A were categorized by SOC, the most frequently reported SOCs were General disorders and administration site conditions (44.12%, 15/34 participants, 23 events), Investigations (29.41%, 10/34 participants, 21 events), and Nervous system disorders and Respiratory, thoracic and mediastinal disorders (each 8.82%, 3/34 participants, 3 events each). According to PT, the most common AEs were Injection site pain (44.12%, 15/34 participants, 16 events), Injection site induration (20.59%, 7/34 participants, 7 events), and Blood creatine phosphokinase increased (14.71%, 5/34 participants, 6 events).

In Part B, the most frequently reported SOCs were General disorders and administration site conditions (68.75%, 11/16 participants, 11 events), Investigations (50.00%, 8/16 participants, 13 events), and Infections and infestations (18.75%, 3/16 participants, 4 events). According to PT, the most common AEs were Injection site discomfort (62.50%, 10/16 participants, 10 events), Blood creatine phosphokinase increased (25.00%, 4/16 participants, 4 events), and Blood bilirubin increased and Upper respiratory tract infection (each 18.75%, 3/16 participants, 5 and 3 events, respectively). Additional SOC and PT categorizations of AEs are presented in Table 4, and ADRs are presented in Table S3.

No participants discontinued the study due to AEs following administration of GB-5001.

### 3.7. Dose Proportionality

Dose proportionality analysis was conducted for the IM administered cohorts: Cohort A (GB-5001A 70 mg), Cohort E (140 mg), and Cohort F (280 mg). Regression analysis was performed to evaluate the relationship between dose and the PK parameters AUC_last_, AUC_inf_, and C_max_. The estimated slopes (95% CIs) were 1.13 (0.91–1.34) for AUC_last_, 1.12 (0.90–1.34) for AUC_inf_, and 1.24 (1.01–1.46) for C_max_, respectively (Table 5). Additionally, differences among dose groups were evaluated using ANOVA, and the results are presented in Table 5.

### 3.8. Pharmacokinetic Modeling and Simulation

In this modeling and simulation, data from participants in Cohorts A, E, and F who received GB-5001A via IM administration were used, while data from Cohort D participants were used for modeling and simulation of oral Aricept^®^ 10 mg. A total of 32 participants were included in the analysis. The dataset incorporated variables such as sex, age, body weight, and plasma concentrations of donepezil. Additionally, CYP2D6 phenotype information was included for participants in Cohorts E and F.

**Figure 7:**
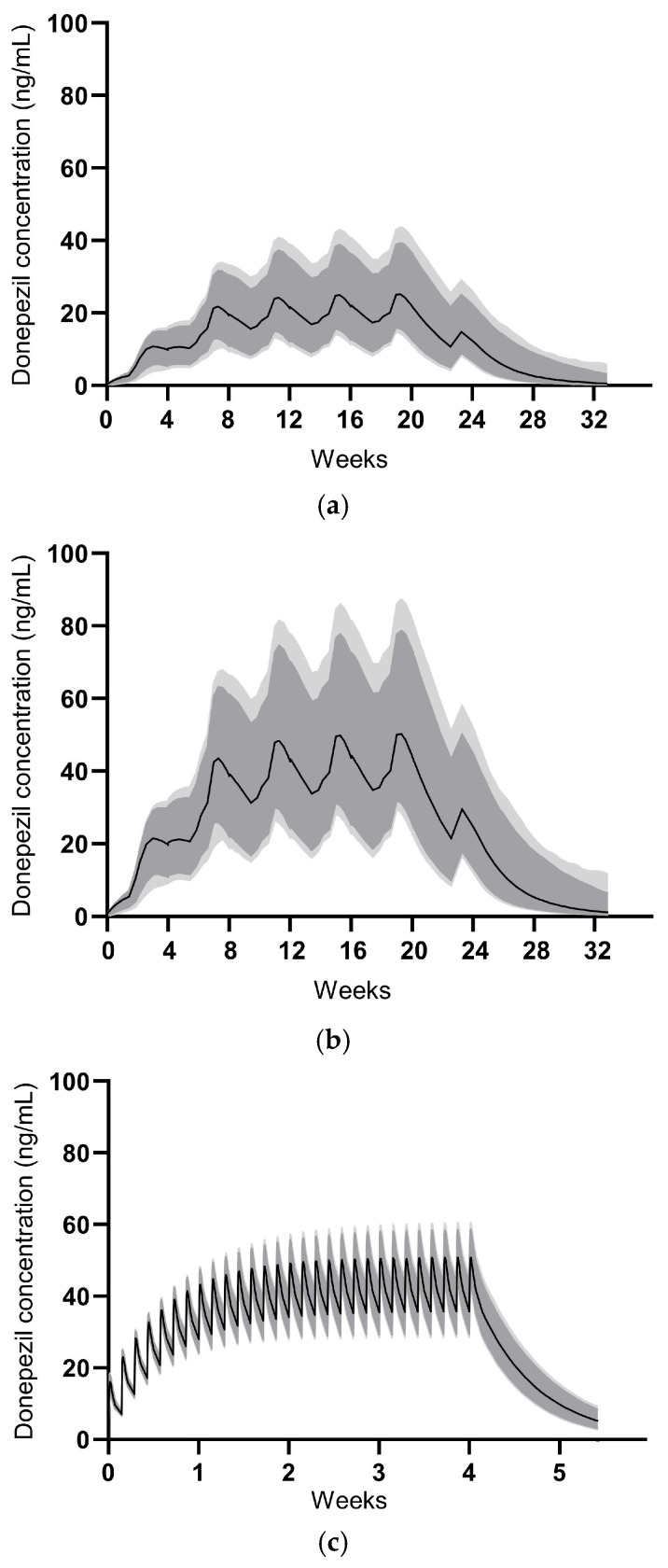
Model-predicted concentrations for GB-5001A multiple administrations. The median predicted concentrations are shown as solid black lines. The dark grey shaded area represents the 90% prediction interval, and the light grey shaded area represents the 95% prediction interval. (**a**) illustrates the profile for GB-5001A 140 mg administered intramuscularly, (**b**) for GB-5001A 280 mg intramuscularly, and (**c**) for Aricept^®^ 10 mg administered orally once daily.

The PK parameters derived from the developed model for GB-5001A, and Aricept^®^ are summarized in Table 6. The PK simulation for GB-5001A was performed assuming IM administration of GB-5001A every 28 days for a total of four doses. GB-5001A plasma concentration values were simulated 1000 times, and the resulting plasma concentration–time profiles along with their 90% and 95% CIs are presented in Figure 7.

The estimate for the central volume of distribution showed relatively high uncertainty (Table 6). This is likely attributable to the flip-flop kinetics characteristic of the LAI formulations, where the absorption phase dominated the concentration–time profile, combined with sparse sampling during the initial distribution phase. Despite this, key exposure parameters were estimated with sufficient precision. The inter-individual variability for clearance and volume terms was observed to be relatively low. This low variability reflects the homogeneity of the study population, which consisted exclusively of healthy male volunteers with strictly defined inclusion criteria. The exclusion of CYP2D6 poor metabolizers in Part B likely further reduced the PK variability associated with donepezil metabolism.

For Aricept^®^ 10 mg, the PK simulation assumed oral administration once daily for 28 days, with 1000 simulations conducted to generate plasma concentration–time profiles and corresponding 90% and 95% CIs, as shown in Figure 7. Simulation-based PK parameters were calculated at steady-state and are presented as mean (coefficient of variation, CV%). The swing_tau was calculated as (C_max,ss_ − C_min,ss_)/C_av,ss_.

For IM administration of GB-5001A 140 mg, the PK parameters were as follows: C_max,ss_ of 26.2 ng/mL (28.0%), C_min,ss_ of 18.3 ng/mL (35.9%), AUC_tau_ of 15,111.5 h·ng/mL (31.5%), C_av,ss_ of 22.5 ng/mL (31.5%), and swing_tau of 0.37 (33.2%). For GB-5001A 280 mg, the PK parameters were: C_max,ss_ of 52.4 ng/mL (28.0%), C_min,ss_ of 36.8 ng/mL (36.1%), AUC_tau_ of 30,259.2 h·ng/mL (31.6%), C_av,ss_ of 45.0 ng/mL (31.6%), and swing_tau of 0.37 (32.5%). For oral administration of Aricept^®^ 10 mg, the PK parameters were: C_max,ss_ of 51.4 ng/mL (8.9%), C_min,ss_ of 36.0 ng/mL (12.2%), AUC_tau_ of 1120.9 h·ng/mL (9.4%), C_av,ss_ of 46.7 ng/mL (9.4%), and swing_tau of 0.33.

### 3.9. CYP2D6

Phenotypic testing for CYP2D6 was conducted exclusively in Part B participants, all of whom were assessed and classified as ultrarapid metabolizer (UM), extensive metabolizer (EM), intermediate metabolizer, or poor metabolizer (PM). In GB-5001A 140 mg IM group, 62.5% (5/8) of participants were classified as EM, and 37.5% (3/8) as intermediate metabolizers; no other phenotypes were identified. In GB-5001A 280 mg IM group, 87.5% (7/8) were extensive metabolizers, and 12.5% (1/8) were intermediate metabolizers, with no participants showing other metabolizer phenotypes.

## 4. Discussion

This study evaluated the safety, tolerability, PK, and PD of GB-5001, a LAI formulation of donepezil, administered via IM and SC routes. In addition, the PK of GB-5001’s metabolite and an exploratory PK profile after multiple dosing were evaluated through modeling and simulation.

The study was designed to enroll 8 participants per cohort, and this target was achieved in all cohorts at the time of IP administration. However, one participant in Cohort F discontinued after dosing, resulting in 7 completers in that cohort, while the demographic characteristics across cohorts remained well balanced, with similar distributions in age and body weight among the healthy male participants. The PK results from this study demonstrated of dose-dependent increases in systemic exposure following IM administration of GB-5001A. Specifically, comparison of dose cohorts receiving GB-5001A 70 mg (Cohort A), 140 mg (Cohort E), and 280 mg (Cohort F) showed consistent increases in key exposure parameters, including AUC_last_, AUC_inf_, and C_max_. This is further supported by the dose proportionality analysis conducted for the GB-5001A IM administered cohorts. Regression analysis indicated that both AUC_last_ and AUC_inf_ increased in a dose-proportional manner following IM administration of GB-5001A. In contrast, C_max_ showed a slightly more than dose-proportional increase, indicating a greater rate of systemic exposure at higher doses. Additionally, ANOVA revealed no statistically significant differences among the dose groups for AUC_last_ (F = 1.57, *p* = 0.2333), AUC_inf_ (F = 2.07, *p* = 0.1538), or C_max_ (F = 2.61, *p* = 0.0985). The observed findings indicate a low likelihood of nonlinear pharmacokinetics within the study dose range, supporting predictable systemic exposure in future clinical settings.

The GB-5001 IM and SC formulations used in this study exhibited delayed absorption, characterized by a gradual increase in plasma concentrations following administration. In contrast to the rapid absorption and elimination observed after a single oral dose of Aricept, the GB-5001A IM and SC formulations and the GB-5001D SC formulation showed a slow rise over approximately 2–3 weeks, followed by a very gradual decline that maintained donepezil concentrations over an extended period. The long-acting depot formulations produced two peaks, which is mechanistically attributable to the characteristics of formulation design. This overall pattern resembles the PK behavior typically seen in flip-flop kinetics, in which the absorption rate is slower than the elimination rate, and absorption becomes the rate-limiting process governing the plasma concentration–time profile. Given that these long-acting formulations form a depot within the muscle or subcutaneous tissue and release the drug slowly over days to weeks, it is likely that the prolonged absorption phase, rather than elimination, predominantly shaped the overall concentration–time curve. The donepezil plasma concentration–time profiles observed in our study therefore suggest the possibility of flip-flop kinetics, and this interpretation is further supported by the markedly delayed T_max_ and the extended apparent half-life observed in the GB-5001 IM and SC groups.

The PK characteristics of GB-5001 suggest absorption-limited, flip-flop kinetics, which have important implications for model development and interpretation. Unlike oral donepezil, which is absorbed rapidly with a high first-order absorption rate constant (ka = 0.7 h^−1^ in our model), the GB-5001 IM and SC formulations exhibited markedly slower absorption with ka values several orders of magnitude lower (KA3 = 0.00173 h^−1^ and KA4 = 0.0113 h^−1^ in our model). GB-5001 also demonstrated two peaks in the concentration–time profile, and this feature was incorporated into the model by applying two delayed absorption rate constants, which was appropriate and well supported during model development. As a result, the terminal phase in the model for the long-acting formulations was governed predominantly by slow depot release rather than systemic elimination. Without accounting for this phenomenon, the model would fail to accurately reflect the pharmacokinetic characteristics of GB-5001. To accurately capture the absorption-limited behavior, the final model of GB-5001A incorporated a depot-based structure consisting of zero-order input and two slow first-order absorption pathways. This approach enabled the model to correctly attribute the extended terminal phase to slow absorption and prevented biased estimation of elimination or distribution parameters.

IM administration of GB-5001A, resulted in a markedly prolonged half-life compared to oral donepezil, reflecting sustained systemic exposure over an extended period. This corresponds to an approximately three- to four-fold increase in half-life, thereby supporting the potential of GB-5001 as a LAI formulation for Alzheimer’s disease.

To further characterize IM absorption, T_lag_ was assessed. In our study, T_lag_ was derived from the time to the first quantifiable plasma concentration. In both the 70 mg and 140 mg IM groups, 7 out of 8 participants had quantifiable donepezil concentrations at the first post-dose sampling time, resulting in a T_lag_ of 0 h for the vast majority of subjects (Table S4). If assay sensitivity or the sampling schedule had been the primary drivers of the observed T_lag_ differences, we would have expected a similar delay to occur in multiple participants within each group. Instead, only one participant per group showed a delayed T_lag_, which may be explained by individual differences in the distribution of muscle and adipose tissue at the injection site and other physiological factors. However, further studies are needed to clarify this finding.

In this study, we also explore the potential impact of CYP2D6 phenotype on the pharmacokinetics of donepezil. In Cohort E, plasma concentration–time profiles were compared between participants classified as extensive metabolizers (EMs) and intermediate metabolizers, according to their CYP2D6 phenotype (Figure S4). Although the EM group showed lower concentrations at earlier time points, their decline in plasma concentrations appeared slower than that of intermediate metabolizers beyond 1512 h. This pattern is generally consistent with previous reports describing differences in donepezil plasma levels according to CYP2D6 genotypes or phenotype [15,16]. However, the sample size was too small to draw any definitive conclusions. In addition, the only intermediate metabolizer identified in Cohort F discontinued the study on Day 64 for personal reasons, resulting in incomplete PK sampling. Consequently, the PK analysis for Cohort F was limited to EM participants. In conclusion, the number of participants was insufficient to enable meaningful statistical or pharmacokinetic comparisons between EM and intermediate metabolizer groups. Therefore, the observation can only be considered a preliminary finding that warrants further investigation in a larger, adequately powered study.

PK analysis of 6-O-desmethyl donepezil revealed that its systemic exposure and peak plasma concentrations were markedly lower than those of the parent compound. These findings were similar to those reported in previous study reporting that plasma concentrations of 6-O-desmethyl donepezil were near or below the lower limit of detection (0.2 ng/mL = 200 pg/mL) [17].

In this study, AChE inhibition (%) was used to evaluate PD response. Since the assessment was conducted in healthy participants, clinical efficacy in patients with Alzheimer’s disease was not evaluated. However, considering previous studies have shown that AChE inhibition of 65% or higher is associated with improvement in Alzheimer’s disease assessment scale-cognitive subscale (ADAS-cog) scores, the level of inhibition observed in this study was approximately 37%, and this degree of inhibition was insufficient to clearly infer clinical efficacy [18]. However, it is noteworthy that the PD effect observed in this study was comparable to that reported following a single oral dose of donepezil in a previous study [19].

No participants discontinued treatment due to AEs, and no serious or clinically significant events were reported. A single Grade 3 increase in blood creatine phosphokinase occurred following GB-5001A IM administration but resolved without intervention. Most ADRs were mild and primarily related to injection sites. Notably, such reactions were absent following intramuscular administration in Part A but present in Part B, despite using the same formulation. This difference is likely attributable to the larger injection volumes required for the higher doses in Part B, suggesting a potential association between injection volume and local tolerability. However, as all injection site-related ADRs resolved without clinical intervention, these findings suggest that GB-5001A is well tolerated when administered via the IM route.

Modeling and simulation results indicated that GB-5001A administered intramuscularly every 28 days reached near steady-state after three doses, with plasma concentrations maintained within a target range thereafter. Notably, GB-5001A 280 mg IM produced C_max,ss_ and AUC_τ_ values comparable to those observed with once-daily oral administration of Aricept^®^ 10 mg, supporting the feasibility of GB-5001A as a long-acting alternative.

Compared to the previous GB-5001 study, the present study demonstrated a lower C_max_ and a delayed T_max_ for GB-5001A [14]. The prolonged T_max_ may indicate a further refinement of the LAI properties of the GB-5001A. Given that donepezil is known to be associated with dose-dependent AEs, the reduced C_max_ observed in this study may contribute to improved tolerability in clinical use [20]. Notably, despite these differences, overall systemic exposure remained comparable between the two studies, supporting the potential of GB-5001A as a viable LAI formulation for future clinical application. However, additional studies are needed before these findings can be applied in clinical practice, as this study was conducted only in healthy male volunteers, whereas donepezil is typically administered to an elderly population of both sexes.

## 5. Conclusions

GB-5001A exhibited a favorable safety and tolerability profile, along with dose-proportional pharmacokinetics and a markedly prolonged half-life compared to oral donepezil. Although C_max_ increased slightly more than proportionally, overall systemic exposure remained consistent across dose levels. These findings indicate that GB-5001A may serve as a viable LAI alternative to daily oral donepezil for once-monthly administration.

## Figures and Tables

**Table 1:** Study participant demographics.

Part A						
	GB-5001A 70 mg IM (*n* = 9)	GB-5001A 70 mg SC (*n* = 9)	GB-5001D 70 mg SC (*n* = 8)	Aricept^®^ 10 mg (*n* = 8)	All (*n* = 34)	*p*-Value
Sex						
Male	9 (100.00)	9 (100.00)	8 (100.00)	8 (100.00)	34 (100.00)	-
Age (years)	29.22 ± 6.82	26.78 ± 4.76	30.25 ± 5.47	32.25 ± 8.19	29.53 ± 6.43	0.3748
Height (cm)	172.37 ± 7.13	172.33 ± 5.54	173.80 ± 7.95	176.23 ± 3.58	173.60 ± 6.20	0.5561
Weight (kg)	74.28 ± 8.90	70.00 ± 7.54	74.74 ± 10.75	74.51 ± 8.25	73.31 ± 8.72	0.6403
Body mass index (kg/m^2^)	25.03 ± 2.88	23.61 ± 2.63	24.70 ± 2.84	23.94 ± 1.97	24.32 ± 2.56	0.6480
**Part B**						
			**GB-5001A** **140 mg IM** **(*n* = 8)**	**GB-5001A** **280 mg IM** **(*n* = 8)**	**All** **(*n* = 16)**	***p*** **-Value**
Sex						
Male			8 (100.00)	8 (100.00)	16 (100.00)	-
Age (years)			30.75 ± 10.21	31.25 ± 6.02	31.00 ± 8.10	0.9067
Height (cm)			172.28 ± 7.27	173.94 ± 5.02	173.11 ± 6.10	0.6029
Weight (kg)			72.69 ± 13.39	70.98 ± 7.21	71.83 ± 10.43	0.7548
Body mass index (kg/m^2^)			24.35 ± 2.98	23.49 ± 2.61	23.92 ± 2.74	0.5480

Notes: Age, height, weight, and body mass index are presented as the mean ± standard deviations. The *p*-values for Part A were derived using analysis of variance (ANOVA), whereas those for Part B were derived using the *t*-test. Abbreviations: Intramuscular (IM), Subcutaneous (SC).

**Table 2:** Pharmacokinetic parameters of donepezil.

Pharmacokinetic Parameters	GB-5001A 70 mg IM	GB-5001A 70 mg SC	GB-5001D 70 mg SC	Aricept^®^ 10 mg	GB-5001A 140 mg IM	GB-5001A 280 mg IM
*n*	8	8	8	8	8	7
C_max_ (ng/mL)	5.28 (4.06, 6.87)	3.78 (2.43, 5.88)	5.23 (2.85, 9.6)	17.54 (15.66, 19.63)	13.71 (10.46, 17.97)	29.12 (23.66, 35.84)
T_max_ (h)	1176.00 (552.00, 1344.00)	1512.00 (768.00, 1512.02)	924.00 (504.00, 1176.00)	2.50 (1.50, 3.00)	816.72 (519.12, 1284.93)	619.19 (442.79, 865.85)
T_lag_ (h)	6.00	1.86 (0.21, 16.34)	0.71 (0.01, 57.80)	-	0.5	-
AUC_inf_ (h·ng/mL)	6326.81 (4841.49, 8267.8)	5653.52 (3907.87, 8178.96)	4414.72 (2115.25, 9213.95) [n = 7]	1078.73 (978.21, 1189.59)	16,803.08 (13,076.75, 21,591.25) [n = 7]	29,693.07 (24,705.24, 35,687.92)
AUC_last_ (h·ng/mL)	6119.81 (4694.41, 7978.01)	4831.61 (3244.52, 7195.02)	4219.61 (2249.72, 7914.38)	927.53 (857.93, 1002.78)	15,523.91 (12,179.46, 19,786.74)	28,981.27 (24,212.79, 34,688.86)
AUC_0–720_ (h·ng/mL)	1773.33 (1282.41, 2452.19)	711.37 (440.19, 1149.63)	1583.34 (777.48, 3224.49)	1073.26 (974.65, 1181.84)	4341.46 (2638.35, 7143.94)	10,028.22 (8342.81, 12,054.12)
CL/F (L/h)	11.06 (8.47, 14.46)	12.38 (8.56, 17.91)	15.86 (7.60, 33.09) [n = 7]	9.27 (8.41, 10.22)	8.33 (6.48, 10.71) [n = 7]	9.43 (7.85, 11.33)
V_d_/F (L)	5016.88 (3953.75, 6365.88)	7587.24 (4433.43, 12,984.56)	6159.31 (3048.22, 12,445.66) [n = 7]	1200.55 (1058.64, 1361.49)	3448.76 (2746.38, 4330.77) [n = 7]	3937.14 (3170.05, 4889.85)
t_1/2_ (h)	314.3 (276.31, 357.52)	424.75 (306.08, 589.43)	269.25 (161.48, 448.96) [n = 7]	89.77 (76.19, 105.77)	286.91 (251.35, 327.51) [n = 7]	289.4 (240.32, 348.52)

Notes: Data are presented as the geometric mean with 95% confidence intervals. T_max_ values are median (minimum, maximum). In the GB-5001D 70 mg SC group, one participant was excluded from the calculation of elimination-phase-related PK parameters (AUC_inf_, CL/F, V_d_/F, and t_1/2_) because the elimination phase could not be reliably characterized. Abbreviations: area under the blood concentration–time curve from time zero to the time of the last quantifiable concentration (AUC_last_), area under the blood concentration–time curve from time zero to extrapolated to infinity (AUC_inf_), area under the blood concentration–time curve from time zero to 720 h (AUC_0–720_), maximum concentration of drug (C_max_), apparent clearance (CL/F), volume of distribution (V_d_/F), half-life (t_1/2_), time to maximum concentration (T_max_), and time prior to the first measurable concentration (T_lag_), Intramuscular (IM), Subcutaneous (SC).

**Table 3:** Pharmacodynamic parameters of GB-5001A 280 mg.

Variables	Values
n	7
E_max_ (%)	37.06 (28.94, 47.44)
AUE_last_ (h·{%})	52,373.11 (41,037.97, 66,839.15)

Note: Data are presented as the geometric mean with 95% confidence intervals. Abbreviations: area under the effect curve from time zero to the time of the last quantifiable activity (AUE_last_) and the maximum observed AChE inhibition (E_max_).

**Table 4:** Adverse events of the study.

System Organ Class Preferred Term	GB-5001A 70 mg IM	GB-5001A 70 mg SC	GB-5001D 70 mg SC	Aricept^®^ 10 mg	GB-5001A 140 mg IM	GB-5001A 280 mg IM
General disorders and administration site conditions	0	8 (88.89), {12}	7 (87.50), {11}	0	5 (62.50), {5}	6 (75.00), {6}
Injection site pain	0	8 (88.89), {8}	7 (87.50), {8}	0	0	1 (12.50), {1}
Injection site induration	0	4 (44.44), {4}	3 (37.50), {3}	0	0	0
Injection site discomfort	0	0	0	0	5 (62.50), {5}	5 (62.50), {5}
Investigations	0	0	1 (12.50), {1}	0	3 (37.50), {3}	5 (62.50), {10}
Alanine aminotransferase increased	0	0	1 (12.50), {1}	0	0	1 (12.50), {1}
Blood bilirubin increased	0	0	0	0	1 (12.50), {1}	2 (25.00), {4}
Blood creatine phosphokinase increased	0	0	0	0	0	4 (50.00), {4}
Cardiac disorders	0	1 (11.11), {1}	0	0	0	2 (25.00), {2}
Arrhythmia	0	1 (11.11), {1}	0	0	-	-
Bradycardia	0	0	0	0	0	1 (12.50), {1}
Tachycardia	0	0	0	0	0	1 (12.50), {1}
Infections and infestations	0	0	0	2 (25.00), {3}	1 (12.50), {1}	2 (25.00), {3}
Upper respiratory tract infection	0	0	0	2 (25.00), {2}	1 (12.50), {1}	2 (25.00), {2}
Nasopharyngitis	0	0	0	1 (12.50), {1]	0	1 (12.50), {1]
Gastrointestinal disorders	0	0	1 (12.50), {1}	1 (12.50), {1}	1 (12.50), {1}	0
Nausea	0	0	0	1 (12.50), {1}	0	0
Vomiting	0	0	1 (12.50), {1}	0	0	0
Abdominal discomfort	0	0	0	0	1 (12.50), {1}	0
Nervous system disorders	0	0	1 (12.50), {1}	1 (12.50), {1}	0	0
Dizziness	0	0	0	1 (12.50), {1}	0	0
Headache	0	0	1 (12.50), {1}	0	0	0
Ear and labyrinth disorders	0	0	1 (12.50), {2}	0	0	0
Tinnitus	0	0	1 (12.50), {2}	0	0	0

Note: Data are presented as the number of participants (percentage), {number of adverse events}. Abbreviations: Intramuscular (IM), Subcutaneous (SC).

**Table 5:** Dose-Proportionality Analysis of GB-5001A Intramuscular Administration Groups.

Linear Regression					
Variable	Parameter Estimate	Standard Error	t Value	*p*-Value	95% Confidence Intervals (Lower, Upper)
C_max_					
Intercept	−3.55	0.54	−6.61	<0.0001	
Dose	1.24	0.11	11.37	<0.0001	(1.01, 1.46)
AUC_last_					
Intercept	3.98	0.52	7.72	<0.0001	
Dose	1.13	0.10	10.80	<0.0001	(0.91, 1.34)
AUC_inf_					
Intercept	4.05	0.52	7.78	<0.0001	
Dose	1.12	0.11	10.65	<0.0001	(0.90, 1.34)
Analysis of covariance					
**Pharmacokinetic parameters**	**Num DF**	**Den DF**	**F value**	***p *** **value**	
AUC_last_ (h·ng/mL)	2	20	1.57	0.2333	
C_max_ (ng/mL)	2	20	2.61	0.0985	
AUC_inf_ (h·ng/mL)	2	19	2.07	0.1538	

Note: Dose proportionality was assessed using data from participants who received IM administrations of GB-5001A at doses of 70 mg, 140 mg, and 280 mg. Abbreviations: area under the blood concentration–time curve from time zero to the time of the last quantifiable concentration (AUC_last_), area under the blood concentration–time curve from time zero to extrapolated to infinity (AUC_inf_), maximum concentration of drug (C_max_).

**Table 6:** Population Parameter Estimates for the Final Pharmacokinetic Model of GB-5001A.

Parameter	Point Estimate	Standard Error	95% Confidence Interval
CL, L/h	9.57	0.57	8.453–10.687
IIV_CL_	0.0945	0.0334	
V_1_, L	58.2	47.9	−35.684–152.084
IIV_V1_	6.97	1.69	
V_2_, L	2050	303	1456.12–2643.88
KA_3_, 1/h	0.00173	0.000141	0.00145–0.00201
KA_4_, 1/h	0.0113	0.00481	0.00187–0.0207
F3	0.657	0.0172	0.623–0.691
F4	0.195	0.0142	0.167–0.223
Q	100 FIX		
D1	497	5.9	485.436–508.564
ALAG3	299	6.97	285.456–312.544
ALAG4	1130	27.7	1075.708–1184.292
ε_1_ (additive), ng/mL	0.0713	0.0207	0.0307–0.112
ε_2_ (proportional) ^a^	0.271	0.0174	0.237–0.305

Notes: The table presents point estimates, standard errors, and 95% confidence intervals for fixed-effect parameters, inter-individual variability (IIV), and residual error terms. Parameters include clearance (CL), central and peripheral volumes of distribution (V_1_ and V_2_), absorption rate constants (KA_3_, KA_4_), bioavailability fractions (F3, F4), inter-compartmental clearance (Q), absorption lag times (ALAG3, ALAG4), and dose duration (D1). IIV is shown for CL and V_1_. Residual variability is modeled using both additive (ε_1_) and proportional (ε_2_) error terms. Q was fixed at 100 L/h during the modeling process. ^a^ ε_2_, a proportional residual error represented as CV. Abbreviations: IIV, inter-individual variabilities which is also expressed in variances (%coefficient of variation (CV), calculated by CV (%) = sqrt(exp(omega) − 1)) × 100; CL, Clearance; V1, Volume of distribution of central compartment; V2, Volume of distribution of peripheral compartment; KA_3_, absorption rate constant of intramuscular depot 1; KA_4_, absorption rate constant of intramuscular depot 2; F3, bioavailability of intramuscular depot 1; F4, bioavailability of intramuscular depot 2; Q, intercompartmental clearance; D1, duration of zero order absorption; ALAG3, absorption lag time of intramuscular depot 1; ALAG4, absorption lag time of intramuscular depot 2.

## Data Availability

Restrictions apply to the availability of these data. Data were obtained from G2GBIO, Inc. and are available from the authors with permission from G2GBIO, Inc.

## Supplementary Materials

The following supporting information can be downloaded at: https://www.mdpi.com/article/10.3390/pharmaceutics17121517/s1, Figure S1: Basic structural model for oral Aricept^®^ 10 mg population pharmacokinetic modeling and simulation; Figure S2: Goodness-of-fit plots for the final pharmacokinetic model of oral Aricept^®^; Figure S3: Visual Predictive Check plot of population pharmacokinetic model; Figure S4. Plasma concentration–time profile of donepezil stratified by CYP2D6 phenotype in the GB-5001A 140 mg administration group; Table S1: Pharmacokinetic parameters of 6-O-desmythyl donepezil; Table S2. Pharmacokinetic parameters of 6-O-desmythyl donepezil; Table S3. Adverse drug reaction of study; Table S4. T_lag_ data of GB-5001A 70 mg intramuscular administration group and GB-5001A 140 mg intramuscular administration group.

## Abbreviations

The following abbreviations are used in this manuscript:

12-lead ECG	12-lead electrocardiogram
AChE	acetylcholinesterase
ADR	adverse drug reaction
AE	adverse event
ANOVA	analysis of variance
AUC_0–720_	area under the blood concentration–time curve from time zero to 720 h
AUC_inf_	area under the blood concentration–time curve from time zero extrapolated to infinity
AUC_last_	area under the blood concentration–time curve from time zero to the time of the last quantifiable concentration
AUC_τ_	AUC over the dosing interval
AUE_last_	area under the effect curve from time zero to the time of the last quantifiable activity
BQL	below the quantifiable limit
CI	confidence interval
CL/F	apparent clearance
CV	coefficient of variation
CYP2D6	cytochrome P450 2D6
C_av,ss_	average concentration at steady-state
C_max_	maximum concentration of drug
C_max,ss_	C_max_ at steady-state
C_min,ss_	minimum concentration at steady-state
E_max_	maximum activity of the drug
IM	intramuscular
IP	investigational product
IRB	institutional review board
ISR	injection site reactions
LAI	long-acting injectable
LC-MS/MS	liquid chromatography–tandem mass spectrometry
PD	pharmacodynamics
PK	pharmacokinetics
PM	poor metabolizer
PT	preferred term
RBCs	red blood cells
SAE/SADR	serious AE/ADR
SC	subcutaneous
SD	standard deviation
SOC	system organ class
Swing_tau	fluctuation index
T_lag_	time prior to the first measurable concentration
T_max_	time to maximum concentration
UADR	unexpected ADR
UM	ultrarapid metabolizer
t_1/2_	half-life
