# Supplementary material for: Pharmacokinetic Evaluation of GB-5001, a Long-Acting Injectable Formulation of Donepezil, in Healthy Korean Participants: Population Pharmacokinetics with Phase 1 Study
Source: Pharmaceutics. 2025 Nov 25;17(12):1517. doi: 10.3390/pharmaceutics17121517 (PMC12736231; doi:10.3390/pharmaceutics17121517)
Supplement: Supplementary file 1 [file pharmaceutics-17-01517-s001.zip › pharmaceutics-3995520-supplementary.pdf]

pharmaceutics

MDPI

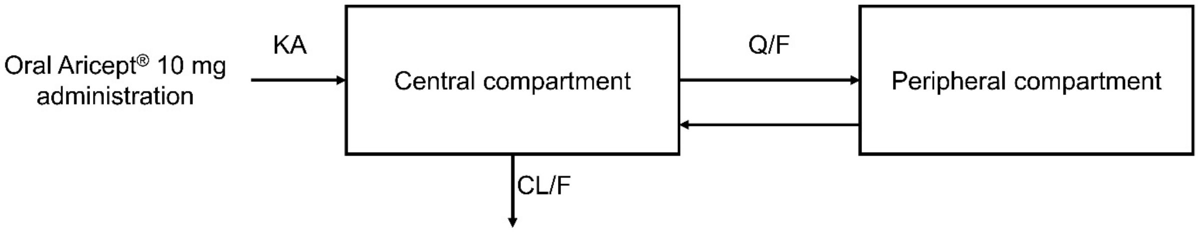

Figure S1. Basic structural model for oral Aricept® 10 mg population pharmacokinetic modeling and simulation. A conventional two-compartment pharmacokinetic model with first-order absorption for orally administered donepezil. In this model, KA represents the first-order absorption rate constant, CL/F is the apparent systemic clearance from the central compartment, and Q/F is the apparent inter-compartmental clearance. Arrows represent the modeled structural flow (absorption, distribution, and elimination) and the associated pharmacokinetic parameters.

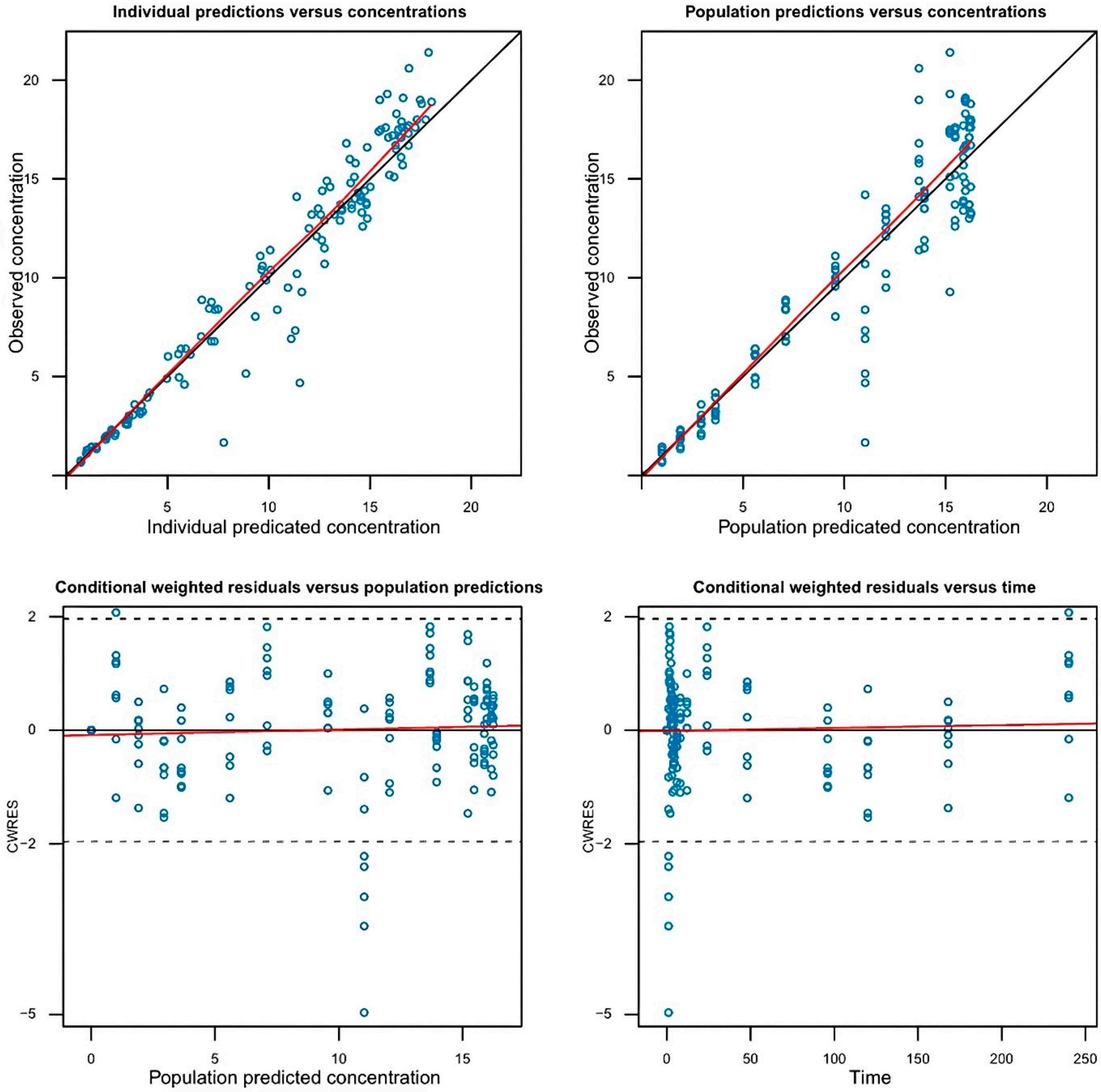

Figure S2. Goodness-of-fit plots for the final pharmacokinetic model of oral Aricept®. The plots display the agreement between observed and predicted concentrations at the individual (Individual predictions versus concentrations) and population (Population predictions versus concentrations) levels, as well as the conditional weighted residuals (CWRES) versus population predictions (Conditional weighted residuals versus population predictions) and versus time (Conditional weighted residuals versus time). The red lines represent locally weighted scatterplot smoothing, and the solid black line in the top panels indicates the line of identity. The horizontal dashed lines represent 2 standard deviations.

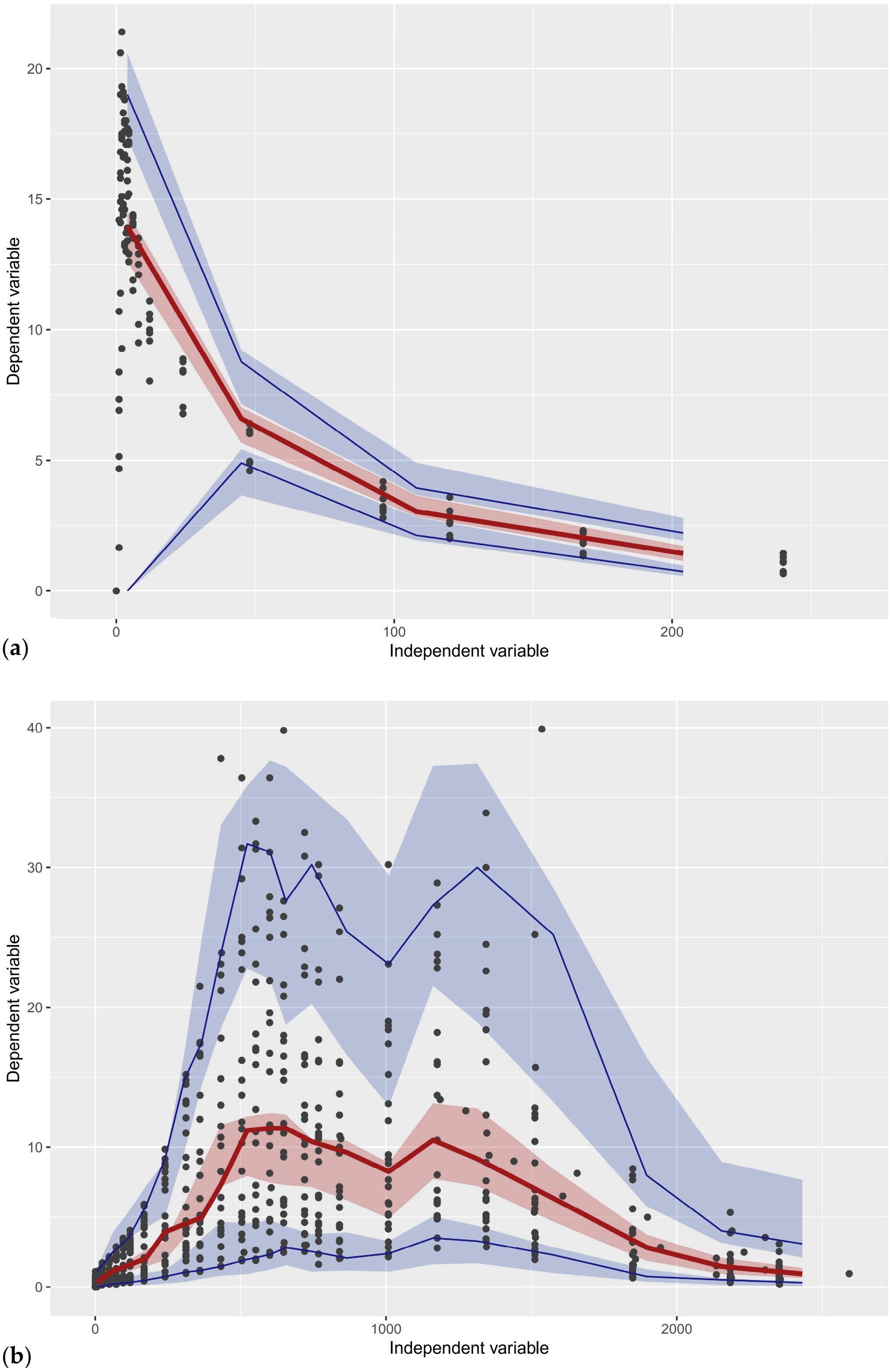

(**a**)

(**b**)

Figure S3. Visual Predictive Check plot of population pharmacokinetic model. This figure illustrates the visual predictive check plot of the population pharmacokinetic model. The black dots represent the observed data. The red line represents the median of the observed values, while the blue lines indicate the 5% and 95% percentiles of the observed values. The red band corresponds to the predicted median interval, and the blue band represents the predicted 5% and 95% intervals. (**a**) Visual Predictive Check plot of final oral Aricept population pharmacokinetic model (N=1000). (**b**) Visual Predictive Check plot of final GB-5001A population pharmacokinetic model (N=1000).

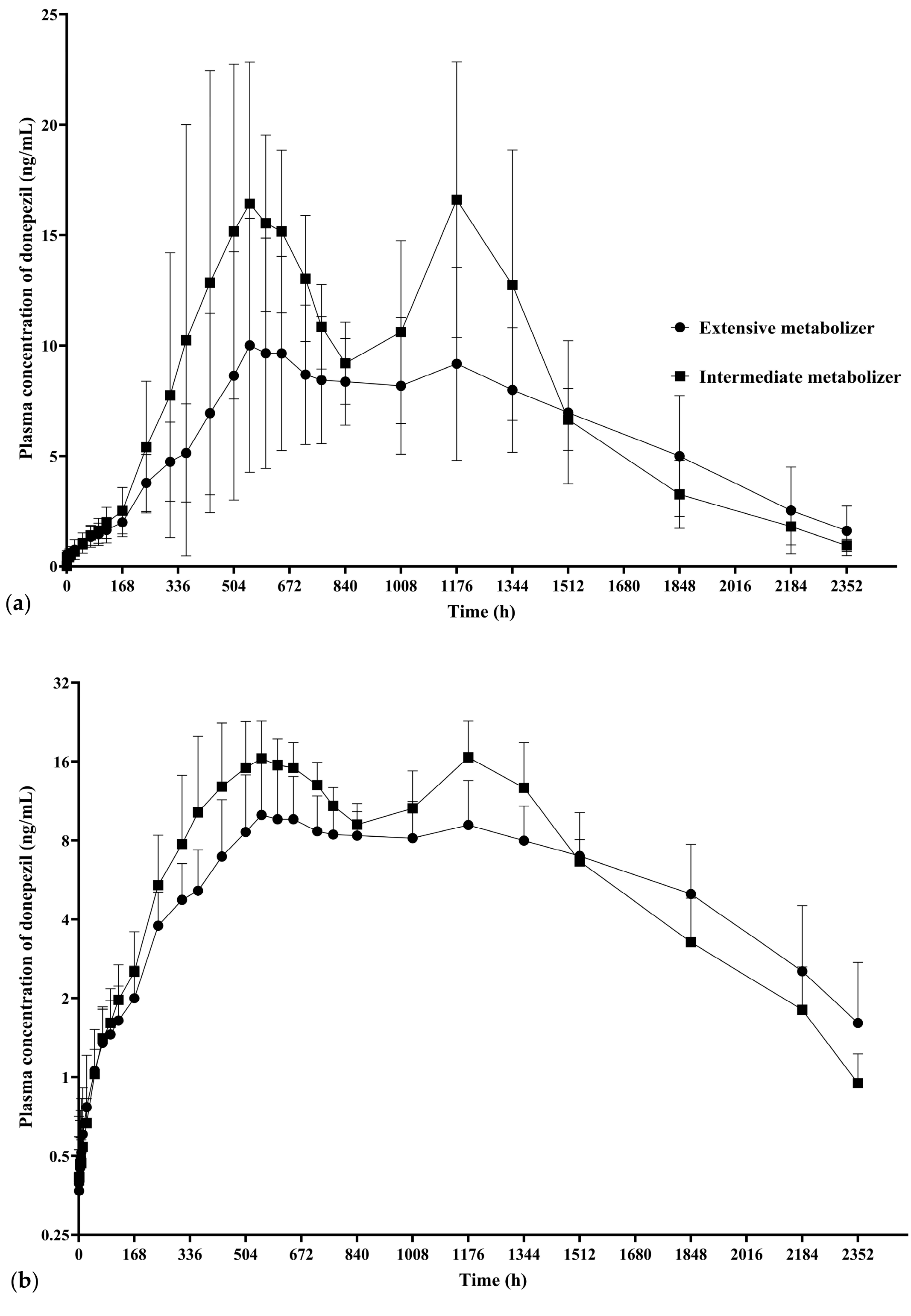

Figure S4. Plasma concentration–time profile of donepezil stratified by CYP2D6 phenotype in the GB-5001A 140 mg administration group. This figure illustrates the plasma concentration of donepezil over time, categorized by CYP2D6 metabolizer phenotype. The curve marked with circles (●) represents the mean plasma concentration for the extensive metabolizer group, while the curve marked with squares (■) represents the mean plasma concentration for the intermediate metabolizer group. The data are present mean ± standard deviation. The inset graph is presented on a semi-logarithmic scale. (**a**) Linear scale. (**b**) Semi-logarithmic scale.

Table S1. Pharmacokinetic blood sampling timepoints.

Cohort	Blood sampling timepoints
Cohort D	pre-dose (0h) and at 1h, 1.5h, 2h, 2.5h, 3h, 3.5h, 4h, 4.5h, 6h, 8h, 12h, 24h (D2), 48h (D3), 96h (D5), 120h (D6), 168h (D8) and 240h (D11) post-dose

Note: Cohort D was Aricept 10 mg oral administration group.

Table S2. Pharmacokinetic parameters of 6-O-desmythyl donepezil.

Parameter	N	Geometric Mean	Geometric Mean 95% CI lower	Geometric Mean 95% CI upper
C_{max} (pg/mL)	7	100.12	66.73	150.20
T_{max} (h)	7	603.96	428.55	851.15
T_{lag} (h)	7	103.70	82.35	130.59
AUC_{inf} (h*pg/mL)	7	85087.25	58899.81	122917.90
AUC_{last} (h*pg/mL)	7	80078.69	54625.31	117392.42
$AUC_{0\text{-}720}$ (h*pg/mL)	7	31156.10	22682.70	42794.84
CL/F (L/h)	7	3290.74	2277.94	4753.84
Vd/F (L)	7	1059461.30	717066.68	1565347.12
$t_{1/2}$ (h)	7	223.16	178.41	279.14

Note: The data were derived from the GB-5001A 280 mg intramuscular administration group. Abbreviations: area under the blood concentration-time curve from time zero to the time of the last quantifiable concentration (AUC_{last}), area under the blood concentration-time curve from time zero to extrapolated to infinity (AUC_{inf}), area under the blood concentration-time curve from time zero to 720 hours ($AUC_{0—720}$), maximum concentration of drug (C_{max}), apparent clearance (CL/F), volume of distribution (Vd/F), half-life ($t_{1/2}$), time to maximum concentration (T_{max}), and time prior to the first measurable concentration (Tlag).

Table S3. Adverse drug reaction of study.

	GB-5001A IM 70 mg (N=9)	GB-5001A SC 70 mg (N=9)	GB-5001D SC 70 mg (N=8)	Aricept® 10 mg (N=8)	GB-5001A IM 140 mg (N=8)	GB-5001A IM 280 mg (N=8)
**System Organ Class** Preferred Term						
General disorders and administration site conditions	0 (0.00), {0}	8 (88.89), {12}	7 (87.50), {11}	0 (0.00), {0}	5 (62.50), {5}	6 (75.00), {6}
Injection site pain	0 (0.00), {0}	8 (88.89), {8}	7 (87.50), {8}	0 (0.00), {0}	0 (0.00), {0}	1 (12.50), {1}
Injection site induration	0 (0.00), {0}	4 (44.44), {4}	3 (37.50), {3}	0 (0.00), {0}		
Injection site discomfort					5 (62.50), {5}	5 (62.50), {5}
Investigations	0 (0.00), {0}	0 (0.00), {0}	1 (12.50), {1}	0 (0.00), {0}	3 (37.50), {3}	2 (25.00), {4}
Alanine aminotransferase increased	0 (0.00), {0}	0 (0.00), {0}	1 (12.50), {1}	0 (0.00), {0}	2 (25.00), {2}	0 (0.00), {0}
Blood bilirubin increased					1 (12.50), {1}	2 (25.00), {4}
Cardiac disorders	0 (0.00), {0}	1 (11.11), {1}	0 (0.00), {0}	0 (0.00), {0}	0 (0.00), {0}	1 (12.50), {1}
Arrhythmia	0 (0.00), {0}	1 (11.11), {1}	0 (0.00), {0}	0 (0.00), {0}		
Bradycardia					0 (0.00), {0}	1 (12.50), {1}
Gastrointestinal disorders	0 (0.00), {0}	0 (0.00), {0}	1 (12.50), {1}	1 (12.50), {1}		
Nausea	0 (0.00), {0}	0 (0.00), {0}	0 (0.00), {0}	1 (12.50), {1}		
Vomiting	0 (0.00), {0}	0 (0.00), {0}	1 (12.50), {1}	0 (0.00), {0}		
Nervous system disorders	0 (0.00), {0}	0 (0.00), {0}	1 (12.50), {1}	1 (12.50), {1}		
Dizziness	0 (0.00), {0}	0 (0.00), {0}	0 (0.00), {0}	1 (12.50), {1}		
Headache	0 (0.00), {0}	0 (0.00), {0}	1 (12.50), {1}	0 (0.00), {0}		
Ear and labyrinth disorders	0 (0.00), {0}	0 (0.00), {0}	1 (12.50), {2}	0 (0.00), {0}		
Tinnitus	0 (0.00), {0}	0 (0.00), {0}	1 (12.50), {2}	0 (0.00), {0}		

Note: Data are presented as the number of participants (percentage) {number of adverse events}. Abbreviations: Intramuscular (IM), Subcutaneous (SC).

Table S4. T_{lag} data of GB-5001A 70 mg intramuscular administration group and GB-5001A 140 mg intramuscular administration group.

**GB-5001A 70 mg intramuscular group**		**GB-5001A 140 mg intramuscular group**	
Participant No.	T_{lag} (h)	Participant No.	T_{lag} (h)
1	0.000	1	0.500
2	0.000	2	0.000
3	0.000	3	0.000
4	0.000	4	0.000
5	0.000	5	0.000
6	0.000	6	0.000
7	0.000	7	0.000
8	6.000	8	0.000
